# Wellness project implementation within Houston’s Faith and Diabetes initiative: a mixed methods study

**DOI:** 10.1186/s12889-020-09167-6

**Published:** 2020-07-02

**Authors:** Rebecca Wells, Ellen D. Breckenridge, Stephen H. Linder

**Affiliations:** 1 Department of Management, Policy, and Community Health, The University of Texas School of Public Health, Houston, USA; 2 Institute for Health Policy, The University of Texas School of Public Health, Houston, USA

**Keywords:** Faith-based organizations, Behavioural change models, Community engagement, Evidence-based programs, Dissemination, Implementation, Capacity building

## Abstract

**Background:**

Faith-based health promotion has shown promise for supporting healthy lifestyles, but has limited evidence of reaching scale or sustainability. In one recent such effort, volunteers from a diverse range of faith organizations were trained as peer educators to implement diabetes self-management education (DSME) classes within their communities. The purpose of this study was to identify factors associated with provision of these classes within six months of peer-educator training.

**Methods:**

This study used the Consolidated Framework for Implementation Research (CFIR) to identify patterns from interviews, observations, attendance records, and organizational background information. Two research team members thematically coded interview transcripts and observation memos to identify patterns distinguishing faith organizations that did, versus did not, conduct DSME classes within six months of peer-educator training. Bivariate statistics were also used to identify faith organizational characteristics associated with DSME class completion within this time frame.

**Results:**

Volunteers from 24 faith organizations received peer-educator training. Of these, 15 led a DSME class within six months, graduating a total of 132 participants. Thematic analyses yielded two challenges experienced disproportionately by organizations unable to complete DSME within six months: [1] Their peer educators experienced DSME as complex, despite substantial planning efforts at simplification, and [2] the process of engaging peer educators and leadership within their organizations was often more difficult than anticipated, despite initial communication by Faith and Diabetes organizers intended to secure informed commitments by both groups. Many peer educators were overwhelmed by training content, the responsibility required to start and sustain DSME classes, and other time commitments. Other priorities competed for time in participants’ lives and on organizational calendars, and scheduling processes could be slow. In an apparent dynamic of “crowding out,” coordination was particularly difficult in larger organizations, which were less likely than smaller organizations to complete DSME classes despite their more substantial resources.

**Conclusions:**

Initial commitment from faith organizations’ leadership and volunteers may not suffice to implement even relatively short and low cost health promotion programs. Faith organizations might benefit from realistic previews about just how challenging it is to make these programs a sufficiently high organizational and individual priority.

## Background

The type-2 diabetes now affecting 13% of US adults [1] is largely preventable as well as manageable for people who develop this condition. Regular physical activity [2] and healthy diets [3] greatly reduce diabetes incidence. Such health behaviours also slow the progression of diabetes [4], thereby reducing the risk of outcomes such as cardiovascular disease and death [2]. However, as in other countries, the rates of healthy behaviours are low in the United States: Only half of US adults engage in recommended levels of physical activity [5], and, diets have been increasing in total calories and in the proportion from unhealthy foods [6].

Despite the importance of healthy eating and exercise to prevent and manage diabetes, as well as medication adherence for those with this condition, only a minority of individuals diagnosed with diabetes receive education on these topics. Almost two decades ago, Lorig and Holman observed that health care providers were the ideal entities for delivering self-management education, but were not well structured to do so [7]. Despite substantial efforts since then to expand provider-based self-management education, as well as referrals to community-based programs, few individuals with or at high risk of chronic diseases benefit from these resources. Even among US adults with health insurance, fewer than 7% of those with diabetes receive education about self-care [8, 9]. Although rates of DSME participation vary regionally, the average is below 15% for all subgroups of insured patients [8, 9]. This may be in part because, even in states requiring health insurance to cover DSME, many payers actually require that patients cover part or all of the cost themselves [10]. Gaps in physician referrals and patient awareness have been cited as additional barriers to DSME use [8].

Faith organizations have high potential for curbing the incidence and impact of diabetes. Regular participation in a religious organization is associated with lower risk for chronic disease and mortality for all ethnic groups [11]. For faith communities, nurturing health reflects deep values of care for physical well-being of others and self as part of spiritual practice [12–15]. Personal relationships with faith organizational leaders as well as with other congregants may also build the trust necessary to share and receive information about personal matters such as health [14]. These dynamics make faith communities well suited to programs such as health self-management education led by congregational members. Peers can be credible models for health behavioural change [7], as they typically share common experiences with participants, and appreciate their norms and constraints [16, 17]. Self-management education groups can provide mutual support for healthy behaviours [16]. Such social capital appears to mediate against poor health outcomes for many groups, including people with low incomes and members of disadvantaged racial and ethnic categories.

Recognizing the potential of faith organizations for health promotion, in early 2016, Houston stakeholders in the international initiative Cities Changing Diabetes chose faith-based strategies as a top priority for their city and formed a workgroup to lead this initiative. A consultant involved in this initiative identified funding to learn about peer support work from a team using this approach in China. Houston stakeholders embraced this possibility. Based on work by the project’s academic partner, Faith and Diabetes was premised on viewing all Houstonians as at risk of diabetes, and did not focus exclusively on those with low incomes [18]. Although the workgroup used such evidence to inform their decisions, it was they and not researchers who oversaw the initiative.

The TMF (formerly Texas Medical Foundation) Health Quality Institute agreed to provide training to faith-based volunteers on the Gateway model of DSME peer education, which entails six weekly, 90-min sessions. In keeping with prior research on self-management education and peer support, the Gateway model entails a certified diabetes educator training lay people how to lead interactive classes in community settings on managing one’s physical and emotional health, as well as navigating how these new behaviours affect life roles [7, 16]. Gateway modules address the six key self-management skills outlined by Lorig and Holman of problem-solving, decision-making, resource utilization, partnering with health care providers, action planning, and tailoring all of these strategies to personal circumstances and preferences [7, 19].

Starting in the winter of 2018, members a number of faith organizations participated in combined peer-educator training to lead Gateway DSME classes for their respective communities [20]. Faith and Diabetes organizers had recruited participants for this training through ministers and other formal faith organization leaders. There were no credentials or screenings required for peer educators. However, many of these volunteers were either health care professionals or lay health educators. Some peer educators had diabetes, although, unlike the norm in some prior peer support programs [16], this was not an expectation in Faith and Diabetes. Many were motivated to lead DSME classes because of family members with diabetes, or general concern about the impact of this disease within their community. Faith organizations were encouraged to send pairs of volunteers to training, who could then co-teach classes for their communities, although in some instances more people or one volunteer per organization participated. Members of the Faith and Diabetes workgroup coordinated the scheduling, provided a venue for the training, meals and refreshments, and organized the follow-up interactions.

Peer educators were encouraged to schedule their first set of DSME classes by the end of their training in how to lead these classes. As the peer educators began providing classes, the TMF Health Quality Institute, the Institute for Spirituality and Health, and the University of Texas School of Public Health, as well as an independent consultant facilitating Cities Changing Diabetes, provided ongoing coaching and held periodic reunions with peer educators to celebrate successes, reflect on lessons learned, and share strategies for addressing challenges.

## Methods

### Conceptual model

The conceptual model employed for this study was the Consolidated Framework for Implementation Research (CFIR) [21]. This is a meta-theoretic synthesis of factors identified from prior research as affecting change implementation across a wide range of contexts, and is intended to be adapted for use in any specific setting. CFIR was chosen based on viewing Faith and Diabetes as a complex intervention whose implementation requires collective action among leaders as well as behaviour change among participants [22, 23].

**Fig. 1:**
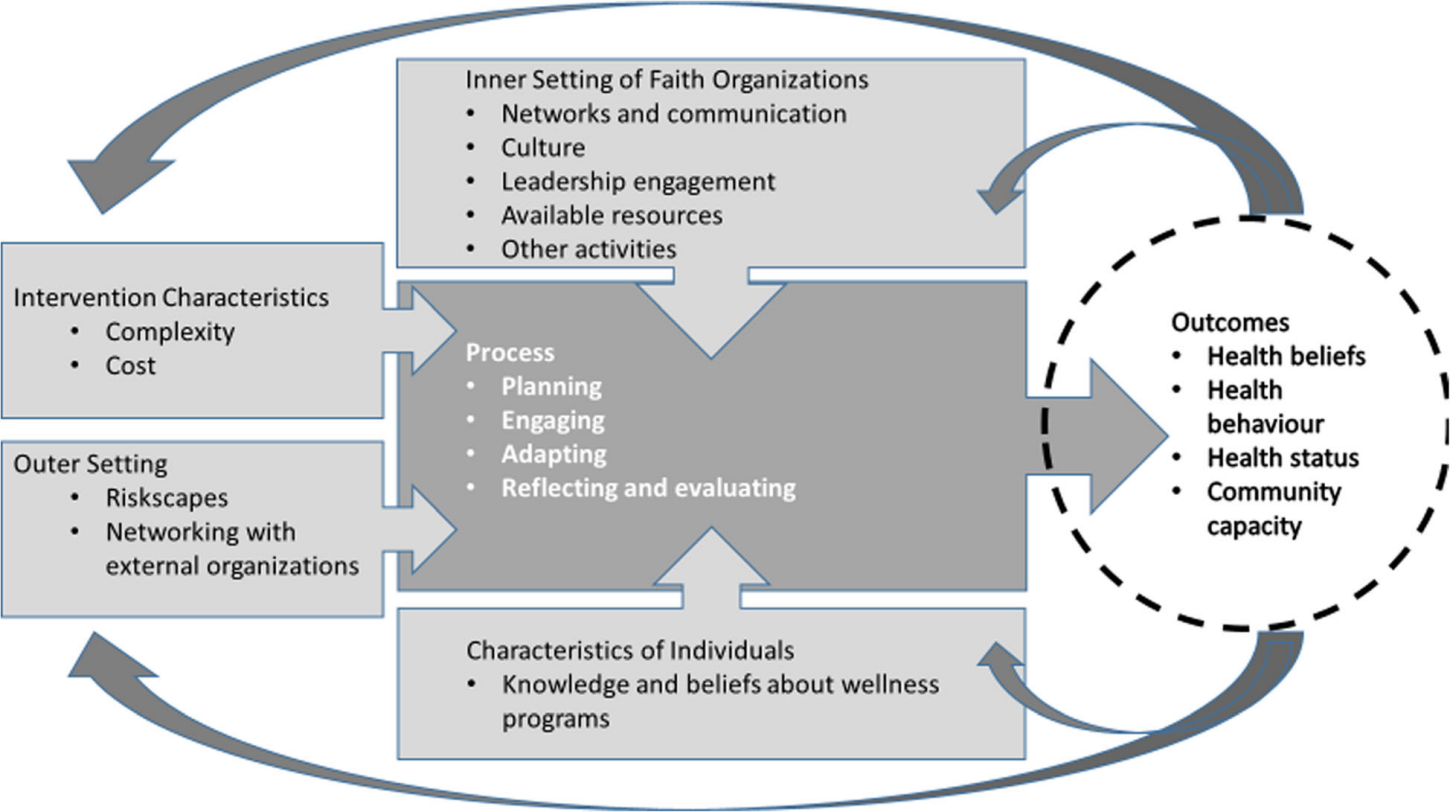
The Consolidated Framework for Implementation Research, as applied to the current study [21]

As shown above in Fig. 1, CFIR entails five categories of factors previously found to affect the implementation of complex process interventions [21, 24]. The first category is Intervention Characteristics. For the current study, we selected “complexity” and “cost” as the most potentially relevant factors, and hence included these among the initial codes. Complexity refers to perceived implementation difficulty, and may be reflected by the intervention’s scope or incompatibility with existing processes [21]. Interventions that require more steps or decisions to implement, thereby become more complex [25].

The Faith and Diabetes workgroup chose the Gateway classes for their relative simplicity, including a free toolkit (a physical box) with a user-friendly facilitation guide for each session, printed handouts for each class participant to keep, and tips on how to prepare class materials with readily available supplies. The Gateway model of DSME was also relatively short, at six sessions, versus 16 specified for the National Institutes of Health-Diabetes Prevention Program [26, 27]. Although Gateway entails very low out-of-pocket costs for peer educators (estimated by the TMF trainer to be below US $20 per set of six classes), Faith and Diabetes provided no financial support for these expenses. Hence, we included an initial code for “cost” to allow for the possibility that peer educators might identify this as an implementation obstacle [21].

The second category of factors addressed in CFIR is the Outer Setting affecting a given intervention’s implementation, such as local socioeconomic patterns or policies [21]. In the current study, the factor originally described as “patient needs and resources” [21] was modified to focus more specifically on “riskscapes.” These refer to local geographic or population concentrations of risk factors for diabetes [28], such as the dependence on automotive transportation and fast food common in Houston. “Networking with external organizations” was also added later as a result of identifying this as salient within Faith and Diabetes.

The third category in CFIR is the Inner Setting, in this instance of faith organizations [21]. Several factors within this category were identified as potentially affecting DSME implementation. The first two Inner Setting factors identified as relevant for the current study were “networks and communication,” including formal and informal communication used for wellness projects within participating faith organizations, and “culture,” defined as the shared norms, values, and assumptions relating to diabetes and wellness promotion among faith organizations and their communities [29]. In addition, we examined three factors related to faith organizational implementation readiness [21]. These were: “leadership engagement,” including championing of Faith and Diabetes activities by ministers and other faith organization administrators [30, 31]; “available resources” for DSME implementation, including money, time, facility space, and training and ongoing coaching [31–33]; and “other activities” complementing or competing with Faith and Diabetes as a relative priority within faith organizations [34]. For instance, participants in prior peer-led DSME have reported missing classes because of competing work and domestic obligations [35, 36].

The fourth category within CFIR is the Characteristics of Individuals involved with implementation, from which “knowledge and beliefs about the intervention,” in this instance, wellness programs such as DSME, was chosen for the current study. Prior research has identified strong peer leadership skills, as well as alignment between their values and programs, as supporting program implementation [37, 38]. Resistance to change, privacy concerns, and information overload have been identified as barriers to participant engagement in peer health education [38].

Finally, from the fifth CFIR category, Process, the current study included initial codes for “planning,” i.e., how realistically and clearly implementation tasks were specified in advance [39];“engaging” participants in Faith and Diabetes activities [30, 40]; and “reflecting and evaluating” by participants on the process of Faith and Diabetes implementation. In addition, although CFIR treats adaptability as an intervention characteristic, we included this construct as the process of “adapting” DSME classes to community needs and norms [41], versus maintaining fidelity to the Gateway model.

### Study design and participants

This study was designed using a stakeholder-engaged approach, including researchers at the University of Texas making study decisions jointly with representatives of the Faith and Diabetes workgroup, composed of faith organizational leaders; the Institute for Spirituality and Health; the TMF Health Quality Institute; the Houston and Harris County Health Departments; and Novo Nordisk, Inc. Analyses began by preparing a summary for each faith organization, including its descriptive profile and DSME implementation timeline [42]. Amalgamated data across faith organizations were then used to identify factors related to completing DSME within 6 months.

### Data collection and measures

Data collection occurred through 1) interviews with Faith and Diabetes key informants and faith organization peer educators, which were transcribed; 2) notes taken during and immediately after observations of peer-educator training and subsequent DSME classes; 3) records of peer-educator attendance in their training; 4) background information, such as faith organizational denomination and size, provided by peer educators or obtained from organizational materials, such as websites; and 5) completion of the Organizational Readiness for Implementing Change questionnaire [43] by peer educators on their final day of DSME training. This questionnaire is a previously validated set of items relating to two dimensions: change efficacy (four items, e.g., ‘We feel confident that we can keep the momentum going in implementing this program’) and change commitment (five items, e.g., ‘We can handle the challenges that might arise in implementing this program.’) [43].

### Analysis

Two members of the research team thematically analysed the qualitative data, beginning with the initial codes described previously based on the Consolidated Framework for Implementation Research [21, 42]. They coded a number of transcripts together, and then continued meeting to review independently coded transcripts and reconcile initial differences in coding. During this time, they also wrote memos on both initial codes that were not salient in the current study context and thus were dropped, such as individuals’ knowledge and beliefs about wellness projects, and dynamics related to initial codes that were salient, such as how other activities competing with DSME classes for time applied to both organizational leaders and participants. The authors then reviewed prior research related to themes in the current study to identify patterns distinguishing Faith and Diabetes from previous faith-based diabetes interventions.

Descriptive statistics on faith organizational characteristics and on comparisons between organizations that did and did not complete DSME classes within 6 months were calculated in Excel and Stata 15.1 [44]. Cronbach’s alphas were used to assess inter-item reliability for the Organizational Readiness for Implementing Change questionnaire scales. Chi-square statistics were used to test for differences between organizations that did and did not implement DSME classes within 6 months of peer-educator training, based on the relative frequencies of characteristics, such as training cohort (first versus second) and size. Student’s t-tests were used to assess differences in peer-educator training attendance and Organizational Readiness for Implementing Change scores, with *p* = 0.05 as the cutoff for statistical significance.

The University of Texas Health Science Center Institutional Review Board reviewed the research protocol and determined that the data collection related to peer-educator training and DSME classes was exempt and waived written informed consent. Individuals who were interviewed provided oral informed consent for these conversations to be taped and de-identified excerpts to be quoted in written materials shared with other cities in Cities Changing Diabetes and the research community.

## Results

### Description of study participants

Forty-one individuals were interviewed: 10 key informants with leadership in Faith and Diabetes as a whole, four faith organization peer educators, and 27 DSME class participants. A total of 56 volunteers from 24 faith organizations completed peer-educator training across two cohorts in 2018–2019.

### Description of faith organizations

As Table 1 shows, 18 of the organizations were Christian (all Protestant), two were interfaith community organizations, one was Muslim, one was Hindu, and one was a secular community organization. In addition, two individuals participated in service of multiple organizations. The secular community organization was a Latino health promotion nonprofit that became involved through a member of one of the participating churches. The majority of organizations were predominantly African-American, and several served Latin American communities.

**Table 1:** Structural characteristics of Faith and Diabetes participating faith organizations

Organizational characteristics	N (%)
Affiliation	
Baptist	7 (29%)
Nondenominational Christian	6 (25%)
United Methodist	2 (8%)
Interfaith community organization	2 (8%)
African Methodist Episcopal	1 (4%)
Hindu	1 (4%)
Nazarenes	1 (4%)
Muslim	1 (4%)
Pentecostal	1 (4%)
Seventh Day Adventist	1 (4%)
Non-religious community organization	1 (4%)
Total (does not =100% due to rounding)	24
Size of faith community	
Fewer than 500 members	10 (42%)
500–999 members	1 (4%)
1000 or more members	8 (33%)
Not applicable (e.g., unaffiliated individuals, non-faith-based organization, or interfaith organizations)	5 (21%)
Total	24

### Description of peer-educator training

The Faith and Diabetes workgroup designed the Cohort I training to include five components: leadership, spirituality, diabetes self-management, approaches to chronic disease prevention, and program evaluation. The leadership component addressed communication skills and strategies for organizations to overcome obstacles and barriers. The diabetes self-management training provided information on adult learning, basic diabetes physiology, and best practices for self-care, while the prevention component included community-based strategies for improving exercise and healthy eating. Training in program evaluation entailed ways to document community experiences with DSME classes and prevention projects so that the communities could assess and refine their efforts as well as demonstrate their effect.

The Faith and Diabetes workgroup included participants from the Institute for Spirituality and Health, TMF Health Quality Institute, the University of Texas School of Public Health, Harris County Health Department, Houston Health Department, the independent consultant for Cities Changing Diabetes, and Novo Nordisk’s Cities Changing Diabetes director for the US. Members of the workgroup met before, during, and after peer- educator training for each cohort, to ensure that the sessions were mutually supportive.

### The role of religion in faith and diabetes

The spirituality component of peer-educator training included asking participants to identify “special things” for their respective faith communities, including practices, foods, and texts related to health. In this respect, Faith and Diabetes training differed from prior peer education training by asking the participants about their own experiences of religious cultures of health, instead of preparing program content in advance around prior assumptions [14, 27, 45, 46]. During these discussions, participants shared cultural norms related to diabetes, although there were no comments in subsequent interviews linking “culture” to whether or not they were able to complete DSME classes. In keeping with prior research [13], some peer educators perceived fatalism about diabetes within their communities. However, comments about faith as a source of motivation and power for healthy behaviours were more common.

So, I mean, I had a lady the other day who came in the May meeting, and she’d been in the hospital for DKA [diabetic ketoacidosis], and she’s very young and a teacher. And the culture of her family is everyone has diabetes ever since her great-grandmother, but nobody has changed their behaviour. So then they get amputations and they die early. *Peer educator*

I’ve seen the results in my own body when I don’t eat animal products and the inflammation goes away. Or I could do the traditional food and use all the medications. Scriptures tells me that I can do all things through the strength of He who sacrificed for me in Calvary. *Peer educator*

In keeping with prior research [47], some peer educators noted traditions of unhealthy foods within their faith communities. For instance, some African-Americans spoke of their traditions of “soul food” such as fried chicken, potatoes and gravy, and sweet breads. All faiths, however, included explicit emphases on the spiritual importance of self-care, such as the Christian belief that the body is a temple for God that should be treated with reverence. Hindu, Muslim, and some Christian participants noted that their traditions included fasting. One of these discussions led to the TMF facilitator following up to look for information about how people with diabetes could safely fast to observe Ramadan. Participants also noted healthy diet and physical activity traditions within their faith communities.

We want to keep our donuts. It’s a special part of our fellowship. *Peer educator*

You can’t change the culture—you can’t tell people not to eat tamales, but you *can* tell them to eat an apple a day. *Peer educator*

Participants from a number of different churches started singing Father Abraham in chorus, clearly from memory. *Notes from training session* ‘Right foot, left foot, turn around, sit down.’ ‘Those are things that we could implement more often … . Because the adults and the children, everyone likes them.’ *Peer educator*

### Factors related to DSME implementation

The TMF trainer encouraged peer educators to schedule their first series of DSME classes by the end of their own training. Thus, in theory, each peer educator was to provide an initial set of six weekly DSME sessions within about 3 months after completing their training. However, the current analysis accommodated delays in class initiation by broadening the time period for completing DSME classes to 6 months. This allowed for a range of possible exigencies, such as seasonal lulls in activity and times when peer educators had to attend to other commitments. Of the 24 organizations, 15 completed DSME classes within 6 months of peer-educator training.

Thematic analyses yielded two overarching themes related to completing DSME classes within 6 months. These were: [1] challenges relating to “complexity,” and [2] challenges “engaging” participants at all levels of faith organizations, from top ministry leaders to community members. Although these are not new themes in peer-education program implementation [17, 30, 31, 35, 41, 48], they played out in some ways not addressed in prior research that have implications for future faith-based health promotion.

### Challenges related to “complexity”

The most salient differentiating factor relative to DSME implementation completion within 6 months of peer-educator training was the Intervention Characteristic of “complexity.” Despite Faith and Diabetes organizers’ efforts, DSME classes were much more complex to implement than anticipated in training, especially for the first Cohort, 47% of which were able to offer completed DSME classes within 6 months (Table 2). These complexities were largely due to training content that peer educators found overwhelming, and difficulties scheduling DSME classes consistently at the right times and places for participants.

**Table 2:** Organizational characteristics (did vs. did not complete DSME classes ≤6 months of peer-educator training)

	Completed 1st set of DSME classes within 6 months of peer-educator training	Did not complete 1st set of DSME classes within 6 months of peer-educator training	Chi square or t-test (Each 1 degree of freedom)	*P*-value
Cohort I, # / total in cohort (%)	7/15 (47%)	8/15 (53%)		
Cohort II, # / total in cohort (%)	8/9 (89%)	1/9 (11%)	4.28	0.04
*For both cohorts combined:*				
Faith organization size				
1000 or more members	2/8 (25%)	6/8 (75%)	7.20	0.01
500–999 members	1/1 (100%), in collaboration with another faith organization	0/1 (0%)	0.63	0.43
Fewer than 500 members	8/10 (80%)	2/10 (20%)	2.24	0.13
Not applicable (*n* = 4) or not available (*n* = 1)	4/5 (80%)	1/5 (20%)	0.83	0.36
Mean % (standard deviation) peer-educator training attendance	96% (6%)	70%	2.79	0.02
Number/total (%) faith organizations scheduling first DSME classes by end of peer-educator training	7/15 (47%)	3/9 (33%)	0.41	0.52
Mean Organizational Readiness for Implementing Change commitment score (standard deviation) (α = 0.84)	5.0 (0.1)	4.8 (0.4)	1.19	0.28
Mean Organizational Readiness for Implementing Change efficacy score (STD) (α = 0.79)	4.9 (0.3)	4.4 (0.8)	1.17	0.13
Number/total (%) faith organization had a budget for wellness programs	4/14 (27%)	1/7 for which information available (14%)	0.53	0.47

Peer educators found that scheduling DSME classes in their faith organizations was also generally difficult, as other programs competed for a few prime times that were feasible for both the peer educators and other community members, and organizational calendars were typically booked months or years in advance. Thus, this aspect of the intervention’s “complexity” entailed managing conflicts with the process dynamics of “other activities.” Peer educators described seeking space that was easy for participants to drive to and find, had free parking, and had space for seating as well as movement. They also sometimes mentioned privacy as desirable, even within their own congregations.

People worry about people knowing about what’s going on with their health and at home, so it’s important to meet off campus or someplace private. We asked if we could meet in [another] building, where few people go, so that the people participating in the workshops would have their privacy protected. We’ve been getting mentoring from the cocaine addiction support group. It’s useful to follow their model, as it is a support and self-help group, and they would never have a meeting on site. … You can’t look at a person and know about mental health or diabetes, so people are often very discrete. The [other building] is several blocks away so that people won’t see if you are driving or walking over to the other site. *Peer educator*

Even organizations whose peer educators had scheduled classes by the end of the training and led those classes within 6 months of training typically had to reschedule some or all of their classes because of unexpected delays. In some instances, space was available at the desired time for one or more weeks, but then pre-empted during subsequent weeks by other faith organizational activities.

Scheduling DSME classes was also particularly problematic for larger faith organizations, categorized for this study as those with more than 1000 members; two of these eight organizations across both cohorts (25%) completed DSME classes within 6 months, significantly below the 63% overall average (Chi square statistic for comparison to all other organizations = 7.20, 1 df, *p*-value = 0.01; Table 2). On the one hand, larger organizations had more available resources for wellness projects, such as full-time administrative staff and prior experience with other wellness initiatives. Large organizations were also sometimes able to field more peer educators; one such notable success story was Taiba, Sisters in Islam. Five leaders from this community co-taught DSME classes that the TMF trainer cited as an exemplar in fidelity to the Gateway model, and their first group of participants graduated within 4 months of the peer-educator training. This team of peer educators has since taught an additional series of these classes.

However, larger faith organizations also had more centralized and formal administrative hierarchies, communications, and scheduling approval processes, which required more lead time for everything from scheduling to advertising classes. Classes were sometimes delayed or cancelled due to denials within these larger organizations, or peer educators never did obtain a six-week slot on the organizational calendar.

At this church, people don’t all know each other the way they do in a small church. … For this project to succeed at (faith organization), someone needs to market it, promote it, and explain why the (faith organization) is doing it. *Faith leader*

The requirement that the training be provided over six consecutive weeks and two hours in duration, presented a challenge to us. My plan was to deliver on Sunday during the one-hour Sunday school sessions, but this would prevent church members who attend other classes from attending. *Peer educator*

### Challenges related to “engagement”

As illustrated by the immediately preceding quote, another common challenge was engaging faith organizational leaders, peer educators, and members of their faith communities for long enough to complete the DSME classes. This combination suggests that, beyond the Inner Setting readiness factor of “leadership engagement” included in the initial codes [21], peer-educator and other congregant readiness to engage was also sometimes insufficient for DSME completion. Of the 233 individuals who enrolled in DSME classes, 132 (57%) graduated (not shown). As in prior faith-based wellness initiatives [49], it was often difficult for organizational leaders to prioritize these classes above many competing demands, suggesting that “engagement” was also related to tensions with “other activities.” One peer educator noted that faith organizations were a logical place to implement such a program, but that engaging the faith community could be challenging.

We are in the Bible Belt and church is the location for big and important parts of civic life. Churches have a powerful ability to modify or change behaviour. I’ve had a hard time getting the strong healing ministry at [my faith organization] to trust Cities Changing Diabetes enough to work with them. They have been very distrustful … . This has made us put a mirror to ourselves and ask how we as a church take advantage of opportunities. *Faith organization leader at organization that did not offer a DSME class within 6 months*.

Both peer educators and members of their communities in smaller as well as larger faith organizations also often struggled to attend the six sessions required by the Gateway model of DSME classes. Two peer educators sought approval from the TMF trainer for their adaptation of the DSME curriculum to fewer than six classes over 6 weeks. One organization spontaneously condensed the last two classes into one because the “group was on a roll”; the peer educator leading the classes subsequently reported their adaptation at a reunion potluck dinner hosted at the Institute of Spirituality and Health. However, the TMF trainer was adamant in her opposition to these deviations from the evidence-based curriculum design, out of concern that a shorter series would be insufficient to support lasting behavioural change.

… if you chose to push for a [condensed] format I will not be able to send you any [DSME class] materials. This curriculum and our contract with CMS [Centers for Medicare and Medicaid Services] are based on evidence-based practices and the research for the Gateway curriculum only shows positive behavioural outcomes using a 6 week format, no less. *Diabetes educator response to peer educators who planned to offer a compressed schedule of DSME classes.*

The preference to adapt the DSME curriculum to fewer, longer sessions stemmed in part from encountering difficulties in securing space for a two-hour class to meet six consecutive weeks at the same place and time.

We found out that many of the hurdles were involved with the church leader … *Peer educator at faith organization that did not offer a DSME class within six months.*

A lot of ‘courting’ has to take place to make this work in churches, because there are lots of competing activities at the church. *Leader at faith organization that did not offer a DSME class within six months.*

Historically, summer attendance is low for our church. Many families schedule vacations, family reunions, and other activities during this time. So, although summer works for my schedule, it does not provide the opportunity to reach potential participants. *Peer educator at faith organization that did not offer a DSME class within six months*.

Just as “complexity” and “engagement” appeared to be the chief challenges facing faith organizations, overcoming these challenges to graduate DSME participants within 6 months of peer-educator training appeared to be related to engaging faith organizational leaders more proactively and reducing the complexity of the peer-educator training.

In keeping with the “reflection and evaluating” Process code, a May 2018 reunion held at the Institute for Spirituality and Health among Faith and Diabetes workgroup leaders, key stakeholders, and Cohort I peer educators prompted a restructuring of the peer-educator training for Cohort II to reduce the level of “complexity.” In Cohort II, peer educators first attended three full-day DSME training sessions on sequential Saturdays in September 2018 and then immediately started scheduling and leading DSME classes at their own faith organizations. Training on prevention projects and evaluation methods were held after DSME was to begin, during January 2019 on two sequential Saturdays. This streamlining of peer-educator training appeared to work: training attendance increased from 79% in Cohort I to 99% in Cohort II, (t-value 3.00, *p*-value = 0.01; not shown). Across both cohorts combined, peer-educator training attendance had been higher among organizations that subsequently completed DSME classes within 6 months (96%), versus among those that did not (70%, t-value 2.79, *p*-value = 0.02; Table 2).

In response to initial “engagement” challenges, the community outreach director at the Institute for Spirituality and Health made concerted efforts to communicate the extent of the organizational as well as volunteer commitment before peer educators began training. She invited additional faith organizations’ ministers and other leaders, not limited to those directly involved in wellness ministries, to an information session to learn about Cities Changing Diabetes and Houston’s Faith and Diabetes initiative. This session emphasized the importance of leadership engagement in DSME classes, the time and space commitment needed for providing these DSME classes to their communities, and the vital role of volunteers serving as peer educators.

### Factors not associated with differential DSME graduation rates

Several factors that prior literature suggested would affect implementation were not related to DSME completion in the current study. These were: The Intervention Characteristic of “cost”; the Inner Setting factors of “networks and communication,” “culture,” and “available resources,” and the Individual Characteristic of “knowledge and beliefs about wellness programs.” Within Outer Setting, “networking with external organizations” emerged as salient, in that peer-educators appreciated the relationships they formed with volunteers from other faith communities, but not a differentiating factor.

“Cost” did not emerge in the interviews as a differentiating factor in interviews for either organizations or individuals. Instead, a few peer educators expressed frustration that volunteers conducting the DSME classes were not being paid for their time and effort, while the people organizing and training peer educators were paid for their part in the initiative. One person trained as a peer educator at the request of her supervisor at work, and then once she began organizing DSME classes at faith organizations, her employer told her she had to take unpaid personal time off for leading those classes.

“Networks and communication” appeared to recognize rather than cause timely DSME completion. Two of the organizations that had completed DMSE classes within 6 months were featured when visitors came from other cities involved in Cities Changing Diabetes: Vancouver; Mexico City; and Leicester, England. This gave peer educators in those organizations opportunities to highlight their successes to an international audience, as well as tell their own leadership about this publicity. Peer educators and other Faith and Diabetes leaders commented in interviews about their sense of pride about being part of a pilot program that might serve as a model for other cities, and they were pleased to have the opportunity to share their experiences and perspectives with others.

I’ve also taken several diabetes courses and this one has been very different because [peer educator] is a little bit more sensitive to our situation, she understands us more … . And the best thing of all is that we’ve taken this class in a friendly environment, with a lot of love—it’s very different. *DSME class participant.*

I learned a lot especially when it was fun to learn, like I told [peer educator] from day one, I've been to the best diabetes class and to be honest they put me to sleep … . it was too structured and I'm like ‘Really?’ *DSME class participant.*

I have a nursing background, so I already was educated a lot about the symptoms and everything. … the classes are working here because [the peer educators] did an excellent job. They made it interesting, they made it tangible, they made it where you would want to do it and come to class. They made the class fun and we had a lot of interaction between each other. That’s what makes the class work. *DSME class participant.*

Relative to “available resources,” faith organizations completing DSME classes within 6 months were not significantly more likely to have budgets for wellness programs than other organizations, although the difference was in the expected direction (27%, versus 14% of those who did not, chi square value 0.53, *p*-value 0.47; Table 2).

Also relating to the roles of Inner Setting factors in implementation, peer educator responses to Organizational Readiness to Implement Change scales were not associated with DSME completion [43]: Peer educators’ responses did not differ between organizations that did versus did not complete DSME classes within 6 months (means of 5.0 versus 4.8, t-value 1.19, *p* value 0.28; and 4.9 versus 4.4; t-value 1.17, *p*-value = 0.13; Table 2). The proportion of organizations scheduling DSME classes by the end of peer-educator training was not significantly associated with subsequent DSME completion (47% among organizations completing DSME classes within 6 months, versus 33% among those that did not; chi square value = 0.41, p-value = 0.52) (Table 2). However, although the differences were not statistically significant, they were in the expected directions (i.e., higher rates of scheduling DSME classes among those subsequently completing such classes within 6 months). Qualitatively, the authors conducting the thematic analyses did not identify differential leadership engagement or knowledge and beliefs about wellness programs between organizations that did versus did not complete DSME classes within 6 months [32].

## Discussion

This study extended research on peer education by examining an initiative that originated from community prioritization, rather than research; was designed to be both situated in and driven by faith organizations [50], with university partners in a supportive role [27]; addressed the health needs of the community as a whole, instead of focusing on the disadvantaged [18, 51]; elicited the roles of faith cultures from peer educators during training and included participants from a range of faith communities in combined peer-educator training cohorts, with ongoing activities to facilitate inter-faith connections [52]. As such, Faith and Diabetes offers a potential exemplar for others around the world seeking to develop locally led health promotion that also build inter-faith social capital potentially applicable to a range of health goals.

One of the key factors related to DSME completion in this study was the Intervention Characteristic of “complexity,” which involved temporal tensions between these classes and “other activities” (Fig. 1). Although complexity is a common theme in implementation [17, 41], we did not find prior studies documenting the dynamics identified in Faith and Diabetes, that: 1) reducing the number of peer-educator training sessions prior to their initiation of DSME classes was associated with a higher rate of successfully completing these classes; and 2) in an apparent “crowding out” phenomenon, larger faith organizations were *less* likely than others to graduate members from DSME within 6 months, despite having more peer educators, a larger number of potential class participants, and overall greater resources.

In addition to “other activities,” these “complexity” challenges intersected with the Process of “adapting.” Most notably, the peer-educators’ need for class scheduling flexibility conflicted with the TMF trainer’s belief about the number of sessions needed to support behavioural change. As with complexity, the tension between fidelity and adaptation is pervasive in program implementation [53]. However, there is little empirical guidance about how to balance these goals while shifting power from elite organizations such as research universities to community organizations such as churches. The complexities of adapting evidence-informed health interventions to faith organizations is further complicated by a lack of mutual awareness between secular and faith leaders in the US. In keeping with prior research [50, 52], in Faith and Diabetes, the fidelity-adaptation tension also co-occurred with very sparse data collection by participating faith organizations, making it impossible to discern whether reductions in the number of DSME sessions extended to impact on participants’ health behavioural change.

In addition, we did not find the challenges of larger faith organizations in prior research [54]. We had expected that larger organizations would be better able to implement DSME classes because of their established health ministries and more prior experience in health promotion [55]; larger numbers of congregants from whom to recruit DSME class participants; greater number of professional staff; and more funds available to cover peer-educator out-of-pocket expenses. In particular, we were surprised to learn that the time frame for securing space for classes could be in years rather than months. Thus, the very organizations with the most resources to support a health ministry seemed to have the most intense competition for logistical support and time. This has significant implications for future health promotion programs, many of which have short-term funding. One prior study found a lower level of diabetes risk reduction program implementation in the larger of two churches, but did not draw conclusions about how size may have affected this divergence [40].

One of the main implications of the current study is to reduce complexity in faith-based wellness promotion wherever possible, with attention to potentially varying challenges across faith organizational size. Even when faith leaders have high motivation to initiate wellness programs, this study implies that they may need long lead times to incorporate them into already full organizational calendars, and multiple levels of internal administrative support to sustain these initiatives. Peer educators may want to consider alternative locations when faith organization facilities are difficult to reserve consistently at times and locations convenient to community members. A related factor is the possibility that even within faith communities, people may want a private space to discuss health conditions.

Realistic previews of the complexities inherent in wellness programs are also important. Faith organization top leadership teams should have full information to decide whether DSME or other diabetes-related wellness projects are a priority, and if so, then advocate for them in the face of competing organizational priorities. Similarly, before even beginning training, peer educators should have realistic information about the time and material costs of the classes they will lead, what can be adapted (e.g., incorporating songs and foods from their tradition) and what cannot (most notably in the current study, the number of DSME sessions), and their faith organization leaders’ engagement.

Despite the apparent importance of some Inner Setting readiness-related factors, in the current study, peer educator perceptions of organizational readiness for change [43] were not associated with DSME completion (Table 2). This may occur in part because of initial overconfidence before peer educators realize how time consuming it will be to coordinate and communicate about the DSME classes. Peer-educators’ faith may also have contributed to the generally high initial readiness estimates, as may socially desirable response bias.

This study had limitations worth noting. Faith and Diabetes differed from many programs in that peer educators did not necessarily have the health condition being addressed themselves. This was a potential limitation because peer educators who did not have diabetes could not speak from their own life experiences about issues such as the emotional fatigue associated with managing the condition over time. However, Faith and Diabetes peer educators did share with DSME class participants core beliefs, culture, and the challenges of incorporating healthy eating and physical activity in a large, car-dependent city. Given the scarcity of qualified peer educators with sufficient time to lead DSME classes, the importance of them having this disease themselves may warrant future exploration. Another limitation of this study is that it did not include the impact of DSME on participants’ health outcomes.

As lifestyle-related diseases take an increasing toll in people’s prospects for living fully and freely, faith organizations continue to offer a powerful potential context for wellness promotion. Faith organizational leaders and external partners will need to make flexible and tenacious long term commitments to actualize the potential of these communities for supporting their members’ health. Although health care access is particularly uneven in the US [56], DSME options vary substantially by location within Europe as well [57]. Religion also plays a prominent role among some immigrant groups in Europe at high risk of diabetes [58]. Hence, findings from the current study about DSME provision within faith organizations has applicability across many countries.

## Conclusions

It has become popular in the United States to suggest faith-based organizations as contexts for public health improvement. The current study both validated and added some caveats to this expectation. Participants deeply appreciated opportunities to learn from and support people from other faiths during peer-educator training. This supports viewing interfaith collaborations as promising for public health improvement. Six months after their training, many peer-educators had offered DSME classes in their communities, and others had not. Those seeking to support faith organizations in wellness programs should help their leaders develop robust plans for implementation, including specific consideration of when and where these programs can be offered, as well as how to demonstrate to congregants that they are a top priority.

## Data Availability

Most of the data for this study are from study participants, who gave written consent for their aggregated group demographic data to be used, and oral consent for deidentified quotations from observations and interviews to be used. In addition, members of the study team also used faith organizations’ publicly available web sites to collect some background information, such as their denomination and size. A de-identified data set is available upon request, and requires execution of a data use agreement with UTHealth. Please contact the corresponding author at Rebecca.S.Wells@uth.tmc.edu.

## Abbreviations

CFIR: Consolidated framework for implementation research
CMS: Centers for medicare and medicaid services
DSME: Diabetes self-management education
